# A porous form Coomassie brilliant blue G250-isorhamnetin fluorescent composite coated with acrylic resin for tumor cell imaging

**DOI:** 10.3389/fchem.2023.1260533

**Published:** 2023-09-18

**Authors:** Jiangpeng Hu, Bo Teng, Zhipeng Xu, Yuanye Wan, Guofan Jin

**Affiliations:** ^1^ Affiliated Peoples Hospital, Jiangsu University, Zhenjiang, Jiangsu, China; ^2^ School of Pharmacy, Jiangsu University, Zhenjiang, China

**Keywords:** isorhamnetin, Coomassie brilliant blue G250, fluorescent complex, acrylic resin, tumor cell imaging

## Abstract

Four distinct fluorescence complexes, the fluorescent complex-1 (FC-1), fluorescent complex-2 (FC-2), fluorescent complex third (FC-3) and fluorescent complex fourth (FC-4), were created using isorhamnetin and Coomassie brilliant blue G250 as raw materials. The issue of isorhamnetin’s low solubility has been resolved, and isorhamnetin-coomassie brilliant blue G250 now has better biocompatibility. Four different forms of fluorescence compounds’ ultraviolet absorption spectra were identified. It was discovered that FC-2, FC-3, and FC-4, respectively, had double peaks at 483–620 nm. FC-4 had the highest ultraviolet absorption intensity, whereas FC-1 exhibited the most consistent and longest wavelength of ultraviolet absorption. Transmission electron microscopy revealed that the acrylic resin evenly disseminated the Coomassie brilliant blue G250-isorhamnetin complex in an amorphous flocculent form. Human prostate cancer cells (PC3) and human cervical cancer cells (HeLa) were investigated in the (Cell Counting Kit-8) CCK8 experiment under 10 different concentration circumstances, and the proliferation impact was 64.30% and 68.06%, respectively. Shown the complex’s strong anti-tumor properties and minimal cytotoxicity. Through *in vitro* imaging of tumor cells, it was found that FC-1’s fluorescent complex has high selectivity and can accurately infiltrate tumor cells, proving that it is biocompatible. The design not only addresses the issue of isorhamnein-Coomassie Bright Blue G250’s bioavailability, but it also has an effective visual fluorescence targeting effect.

## Introduction

**FIGURE 1:**
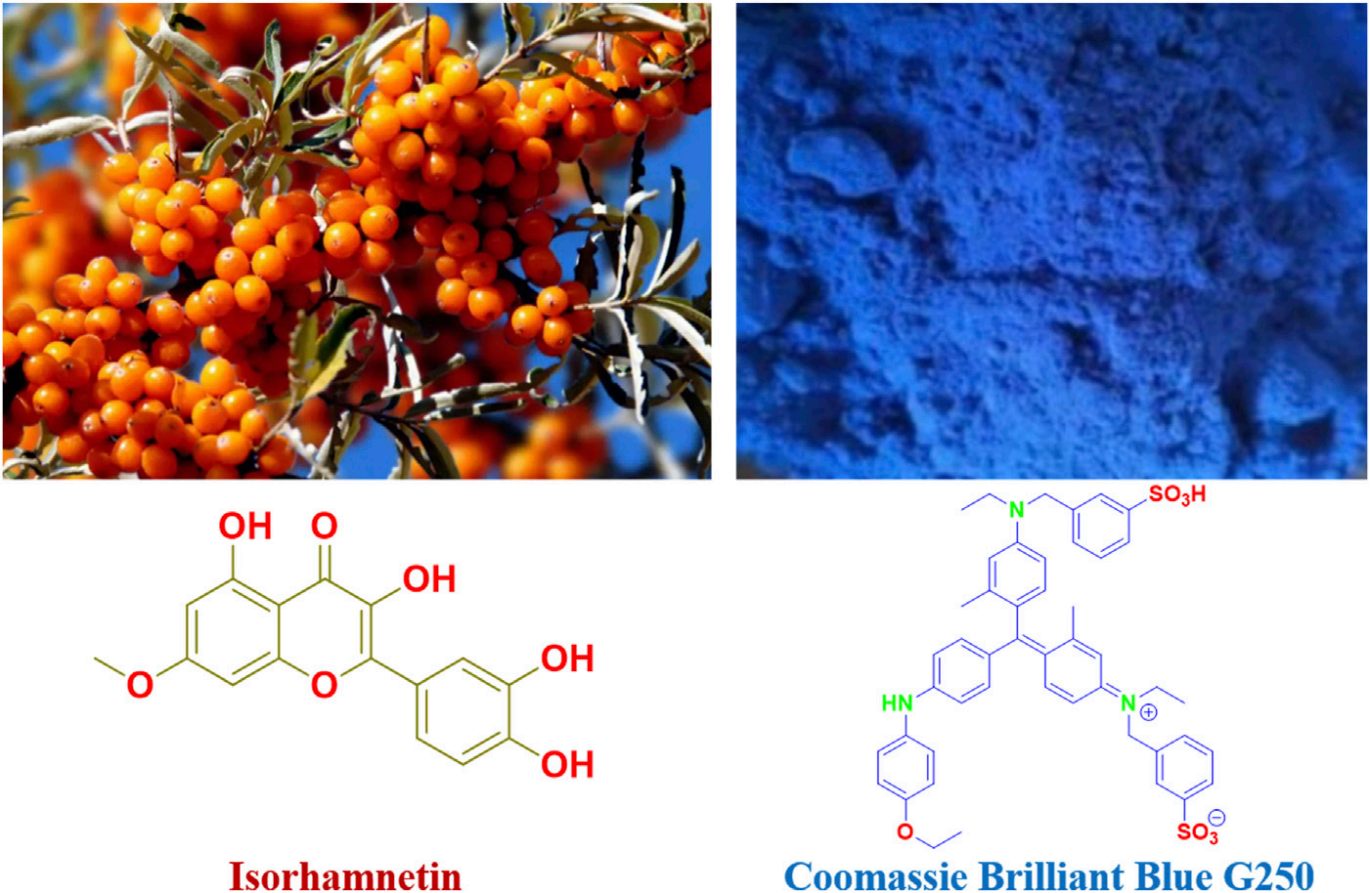
Isorhamnetin and Coomassie brilliant blue G250 structures.

Flavonoids, whose fundamental parent nucleus is 2-phenylchromogen, are a class of chemicals created by joining two benzene rings with phenolic hydroxyl groups through the middle three carbon atoms. In the structure of flavonoids, phenolic hydroxyl, methoxy, methyl, isopentenyl, and other functional groups are often linked. In general, the term “flavonoids” refers to a group of chemicals created by joining two benzene rings together with three carbon atoms. In practically all green plants, flavonoids are present. It is mostly found in higher plants and is involved in a variety of biological processes. For instance, silymarin has liver protection properties, isorhamnetin has antioxidant and anti-tumor activities, baicalin has antibacterial and antiviral actions, total ginkgo biloba flavonoids have therapeutic effects on cardiovascular disorders, and so forth (Li et al., 2021). Isorhamnetin is a kind of flavonoid molecule with excellent antioxidant effects that can prevent aging-related cell and tissue damage (Li et al., 2018; Zhao et al., 2020; Alakhdar and Abou-Setta, 2021). From medicinal plants including ginkgo biloba and sea buckthorn, isorhamnetin (3, 5, 7-trihydroxy-2 -(4-hydroxy-3-methoxyphenyl) benzopyrano-4-one) (Figure 1) is extracted and purified (Long et al., 2017; Ragheb, et al., 2021; Ibrahim, et al., 2021; Long, et al., 2017; Li, et al., 2019). It has a wide range of biological and pharmacological effects, including those that are anti-tumor, anti-viral, anti-myocardial hypoxia, serum cholesterol reduction, anti-allergy, anti-inflammatory, antioxidant, and immunological function regulation. Existing research has demonstrated that isorhamnetin has anticancer effects on a range of tumor cells and clearly inhibits the production of melanin and the control of tyrosinase activity. To induce apoptosis, it is primarily through the PI3K-Akt-mTOR pathway that tumor cell proliferation is inhibited, apoptosis suppressor gene bcl-2 expression is decreased, and pro-apoptotic protein Bax levels are increased (Sanchez et al., 2007; Ma and Chen, 2020; Long et al., 2023).

According to Rui et al., 2023, Liu et al., 2022a; Ming-Yong et al., 2023, the Coomassie Brilliant Blue G250 (Figure 1) is a dye from the triphenyl methane class that is a member of the Coomassie dye family. The interaction between the amino and carboxyl groups in the protein causes Coomassie Brilliant Blue G250 to form a potent, non-covalently bonded complex with the protein (Jianpeng et al., 2022; Nagata et al., 2022; Tingyu et al., 2022; Walied et al., 2023). The dye’s negatively charged anions, such as sulfonic acid groups, are stabilized when a protein-dye combination forms, giving the film or adhesive its noticeable blue hue. For instance, the structure of Coomassie Brilliant Blue G250 combines proteins with hydrophilic characteristics and van der Waals forces via its six benzene ring groups and two sulfonic acid groups.

Acrylic resin has been the subject of substantial study and application, particularly in the field of pharmacy (Wang et al., 2021; Xu et al., 2021). For many medications, insoluble coating technology offers a practical solution. Drugs with limited solubility may become more soluble by coating technology, improving their relative bioavailability in the body and allowing for improved absorption. Additionally, acrylic resin has the benefit of having less hazardous side effects and minimizing bodily harm (Liu et al., 2022b; Ying et al., 2022).

The examination of isorhamnetin’s action *in vitro* and *in vivo* has been severely constrained by its poor solubility; in particular, the uncertainty of concentration in the late one-time pharmacodynamic evaluation makes it challenging to be extensively applied. In addition, it is impossible to determine how selectively the medicine acts in the target cell or whether it is reaching the desired location, among other things. The goal of this project is to maximize the high protein fluorescent dye effect of Coomassie bright blue G250, the high swelling of acrylic resin, and the high activity of isorhamnetin in order to address their low solubility and fluorescence selectivity and achieve the complementary and synergistic effects between them. The breakthrough came from the high activity of isorhamnetin and the capacity of Coomassie Bright Blue G250 to stain proteins when combined with the features of the aforementioned study. By coating with several kinds of acrylic resins, an excellent fluorescence-effect anticancer fluorescent chemical medication was created.

## Experiment

### Materials and instruments

All solvents and reagents were purchased commercially and used without further purification. The reagents used are isorhamnetin (98%, RG) and coomassie bright blue G250 (98%, RG), which were purchased through commercial channels such as Titan Technologies. Full-wavelength absorption spectra were recorded using a UV-2550 spectrophotometer. All optical measurements were performed at room temperature. The transmission electron microscope (TEM) adopts the FEI G2F20 model equipment provided by the United States FEI Company.

### Synthesis

Isorhamnetin (0.2 g, 0.6 mmol) and of potassium hydroxide (0.14 g, 2.5 mmol) were dissolved in EtOH (3 mL) at the room temperature stirred for 1 h, and then Coomassie Brilliant Blue G250 (1.0 g, 1.2 mmol) at the room temperature stirred for 1 h. The EtOH was concentrated to afford 1.3 g of complex.

Isorhamine-coomassie bright blue G250 complex 50 mg was reacted with L100-55, RPO, RS and RL = 100 mg, respectively, in ethanol 3 mL for 3 h. The EtOH was concentrated to afford 30 mg of FC-1.

The FC-2, FC-3 and FC-4 were obtained by the same method.

### Spectroscopic properties

#### UV spectrum

Using a quartz cuvette with a 1 cm path length, ultraviolet-visible (UV-vis) spectra were captured using a UV-2550 spectrophotometer. To create the necessary ideal concentration gradient for later usage, the 10 mg fluorescent complex was dissolved in the following substances: methylene chloride (MC), ethyl acetate (EA), methanol (MeOH), ethanol (EtOH), dimethyl sulfoxide (DMSO), and tetrahydrofuran (THF). According to requirements, spectral tests of solutions at various concentrations were produced, and results were gathered. 400–700 nm is the range of UV-Vis wavelengths. The complex’s fluorescence was measured at an optical path of 10 mm, an excitation wavelength of 450 nm, and an emission wavelength range of 400–800 nm.

### Cellular uptake and localization by transmission electron microscope

A Zeiss Ultra Plus was used for the transmission electron microscope (TEM), which was run at a 15 keV accelerating voltage with an Oxford Instruments X-Max 60 mm^2^ SDD X-ray microanalysis system connected. A silicon wafer was then placed to the sample’s ethanol-suspended precipitate, and the sample was fastened to a sample tray using conductive glue. Using a scanning electron microscope, TEM pictures were then captured at 2.0 m and 200 nm rulers, respectively. A thin backing film is first applied to the copper net, and then the tiny beaker is filled with the appropriate amount of powder and tetrahydrofuran. Ultrasonic oscillation is then performed for 10–30 min. The homogenous mixture of powder and tetrahydrofuran is sucked by a glass capillary tube after three to 5 minutes, and two to three droplets of the mixture are then deposited onto the copper net and dried. Tetrahydrofuran should be volatilized as much as possible by waiting more than 15 min. Last but not least, place the sample on the sample table and place it inside the electron microscope to be examined.

### Cell proliferation toxicity test (CCK8)

Cell pretreatment: In the logarithmic growth phase, PC-3 and Hela cells were digested with trypsin, converted into cell suspension, and the concentration of the cell suspension was adjusted. 5,000 cells per well in 96-well plates were infected, and each group received three different wells. Treatment for cell dosing: samples were given to each experimental group’s cells: six samples from the blank control group (which received no samples) and 16 g/mL were cocultured with the cells for 24 h. It should be observed that the liquid in the outermost circle of the 96-well plate is readily volatilized, leading to the hole drying. This circle should be filled with 150 L of PBS. After 24 h, remove the cells, fill each well with 10 L of CCK-8 solution, and incubate for 2 h in the incubator. Be cautious not to blow bubbles into the wells as this will make it difficult to read the OD value. Measure the absorbance at 450 nm with a microplate reader, save the information, and enter it into the analysis program. Work out the formula.

$$\text{Proliferation }\%=\left(\text{experimental}-\text{empty}\right)\;/\;\left(\text{control}-\text{empty}\right)$$

$$\text{Inhibition }\%=1-\left(\text{experimental}-\text{empty}\right)\;/\;\left(\text{control}-\text{empty}\right)$$

### Cell imaging

Trypsin treatment was applied to HeLa cells in the logarithmic growth phase before they were planted in a 96-well plate with a circular cover, incubated on 5% CO_2_, and cultivated at 37°C for 24 h to promote adhesion. The produced polymer FC-1 stock solutions (20 mg/mL) were made in DMSO, and the relevant amounts of solution were made by diluting them with DMSO. In each sample, the cells in the original culture medium were taken out and replaced after 24 h with a medium containing 20 g/mL. It was then removed with PBS, given two washes, and fixed with paraformaldehyde for 25 min. The procedure involved removing the repair solution with PBS and washing twice, incubating the cells for 20 min in a DAPI-free environment, discarding the staining solution, washing twice with PBS, treating the cells with scaffolds that inhibit fluorescence, and then obtaining fluorescent pictures of the cells under a fluorescence microscope.

## Results and discussion

### Design and synthesis

**SCHEME 1:**
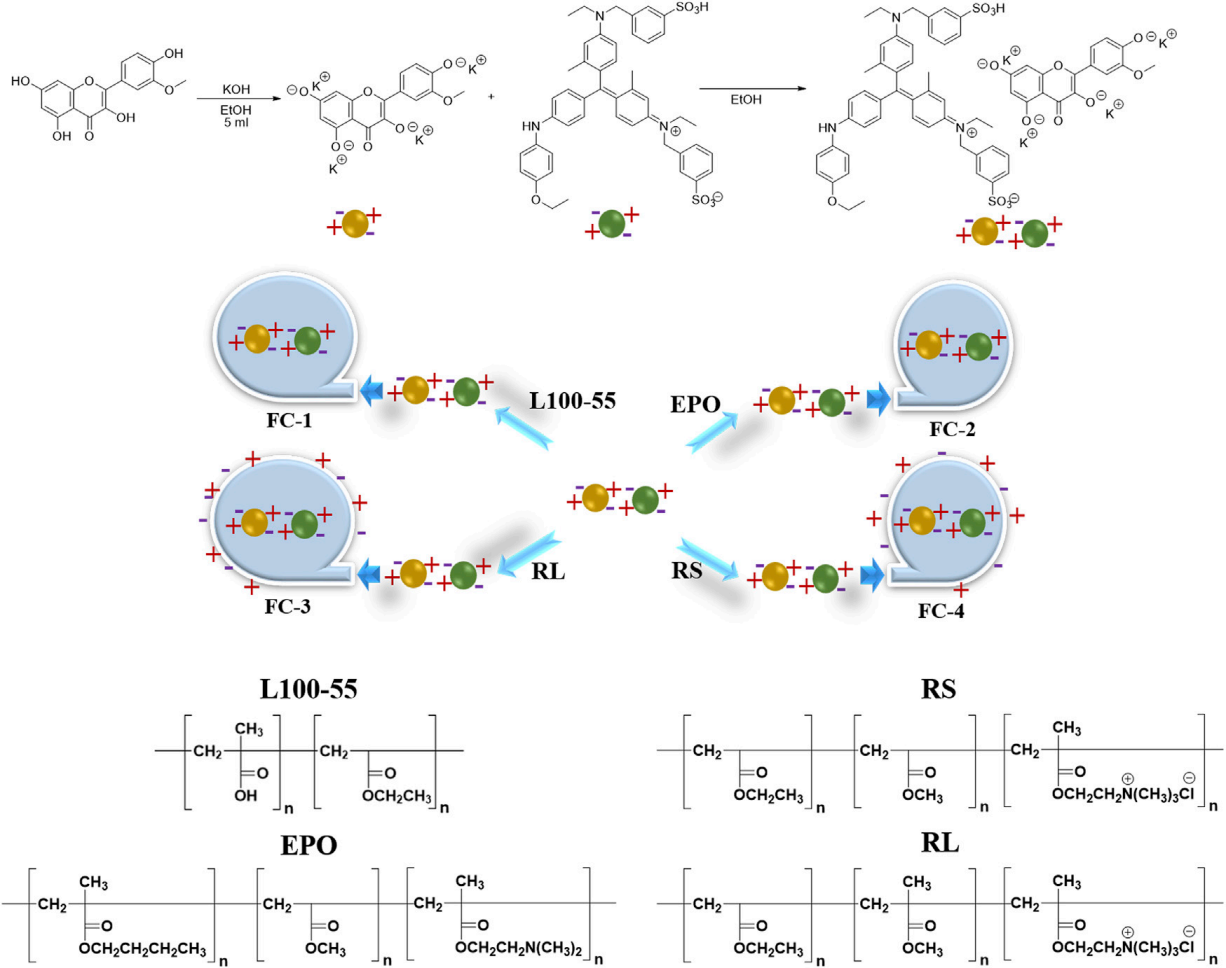
Preparation routes of four fluorescent complexes.

The continuous technique is mostly used to prepare the scheme in the synthesis design area: first, using ethanol as the solvent, potassium hydroxide is used to treat isorhamnetin. To address the solubility issue, all four hydroxyl groups are converted into potassium salt, and zwitterion small molecules are produced. Secondly, the mixing reaction with Coomas Brilliant Blue G250 involves the ion exchange of potassium ions with sulfate ions or oxygen ions with ammonium ions. Finally, to create a porous fluorescent complex, polyionic compounds are covered with various types of acrylic resins to create oil-in-water (Scheme 1).

### Photophysical properties

**FIGURE 2:**
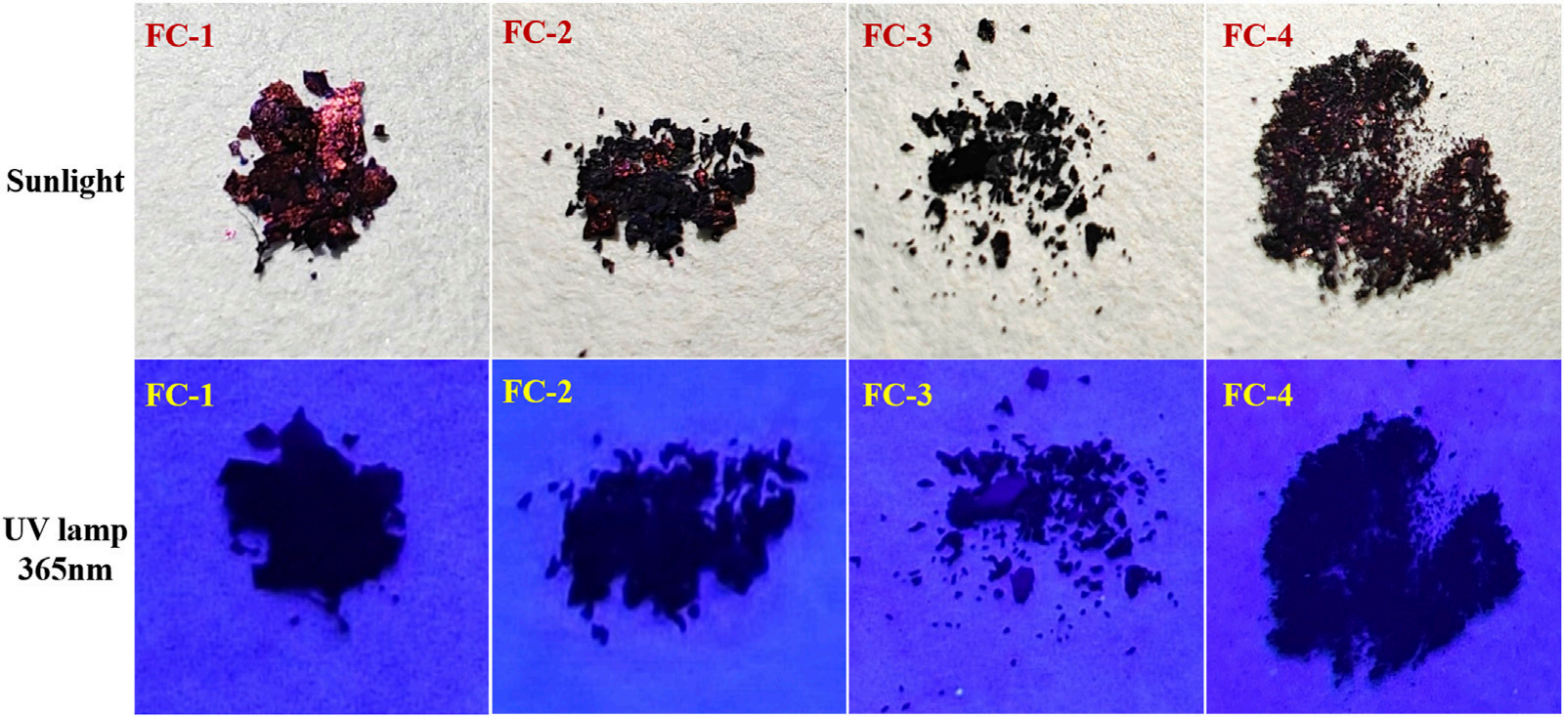
Comparison of four fluorescent complexes under sunlight and 356 nm irradiation.

The four fluorescent compounds produced by the aforementioned methods of production were contrasted in terms of color at 356 nm and natural light. Except for the stunning dark red brightness of FC-1 under direct sunlight, as seen in Figure 2, the other complexes displayed dim or dark color forms that were nearly black at 365 nm.

**FIGURE 3:**
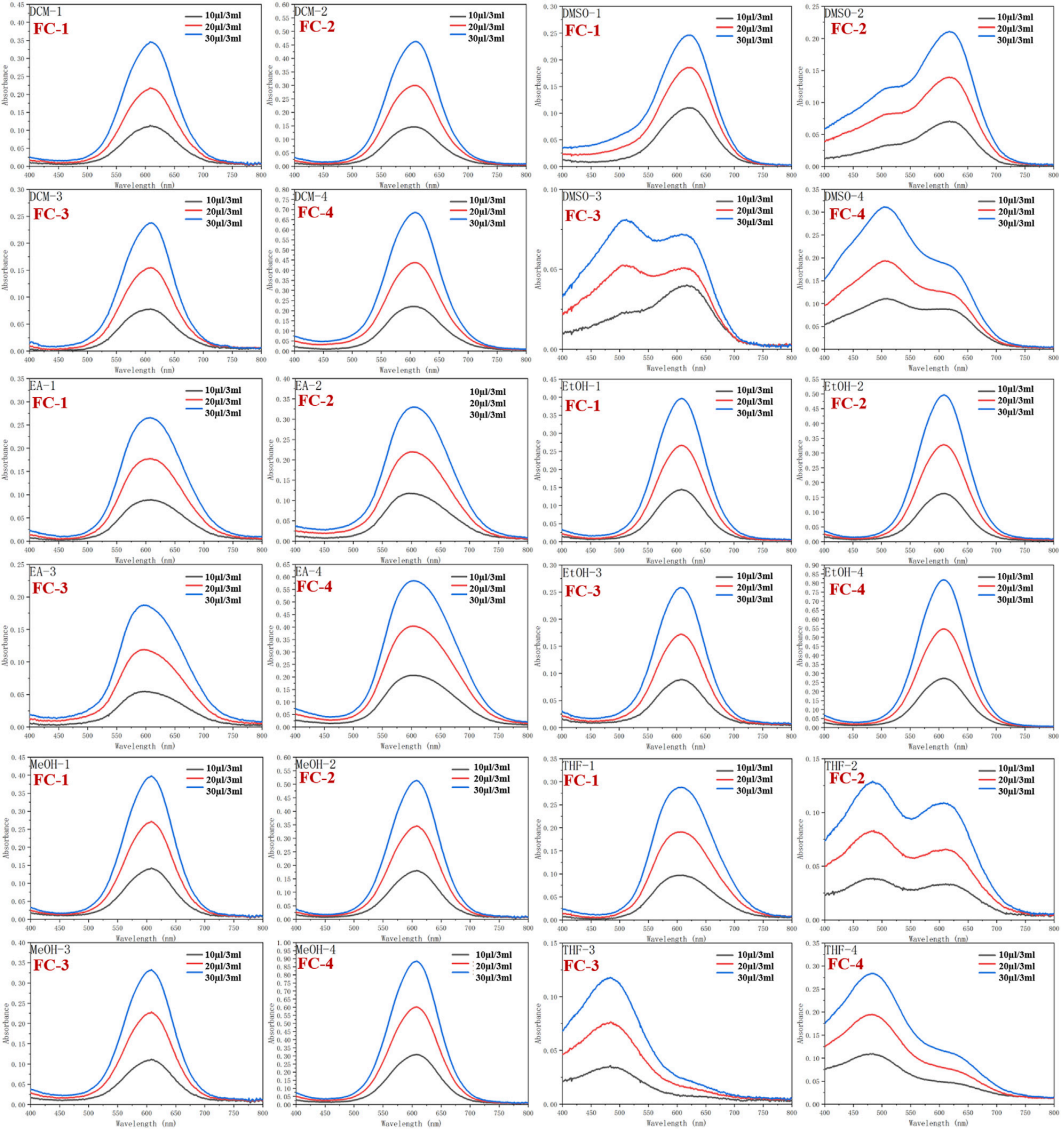
Ultraviolet absorption of four fluorescent complexes in different solvents and concentrations.

Dichloromethane (MC), dimethylsulfoxide (DMSO), ethylacetate (EA), ethanol (EtOH), methanol (MeOH), and tetrahydrofuran (THF) were used as solvents to examine the UV spectra of the four fluorescent complexes. The findings demonstrate that FC-2 and FC-3’s UV absorption spectra in tetrahydrofuran and dimethyl sulfoxide exhibit the identical double peaks in the range of 483–611 nm, respectively, and that the maximal absorption wavelength is in the range of 608–611 nm. This is because different characteristic peaks arise as a result of the amphoteric binding of isorhamine and Coomassie Brilliant Blue G250 in tetrahydrofuran and dimethyl sulfoxide solvents. Because the zwitterion coating form of acrylic resin RS has a superior UV absorption effect than other kinds, the absorption strength of FC-4 in various solvents is two to three times higher than that of other complexes. The FC-1 fluorescent complex has the best UV absorption performance among the four fluorescent complexes. The findings demonstrate that in various solvents, the lowest and maximum absorption wavelengths are 607 and 624 nm, respectively. Additionally, the distinctive peaks in various solvents reveal a smoother and more stable state than other compounds. The absorbance values of the four fluorescent complexes also grew as the concentration did (Figure 3; Table 1-3).

**TABLE 1:** Ultraviolet absorption of four fluorescent complexes in different solvents at 10 μL/3 mL.

Compound	Items	Solvents (10 μL/3 mL)					
		DCM	DMSO	EA	EtOH	MeOH	THF
FC-1	λ_abs_/nm	608	624	610	608	608	611
FC-2	λ_abs_/nm	606	618	596	607	608	483/611
FC-3	λ_abs_/nm	610	617	594	608	608	484
FC-4	λ_abs_/nm	606	509/619	605	608	608	479/620

**TABLE 2:** Ultraviolet absorption of four fluorescent complexes in different solvents at 20 μL/3 mL.

Compound	Items	Solvents (20 μL/3 mL)					
		DCM	DMSO	EA	EtOH	MeOH	THF
FC-1	λabs/nm	608	622	608	609	608	606
FC-2	λabs/nm	607	617	600	608	608	486/611
FC-3	λabs/nm	609	505/608	597	607	608	484
FC-4	λabs/nm	607	507/620	602	608	608	483/619

**TABLE 3:** Ultraviolet absorption of four fluorescent complexes in different solvents at 30 μL/3 mL.

Compound	Items	Solvents (30 μL/3 mL)					
		DCM	DMSO	EA	EtOH	MeOH	THF
FC-1	λabs/nm	608	619	607	608	608	607
FC-2	λabs/nm	608	619	603	609	608	484/607
FC-3	λabs/nm	611	512/608	597	607	608	484
FC-4	λabs/nm	609	504/620	605	608	608	484/618

### Scanning transmission electron microscope

**FIGURE 4:**
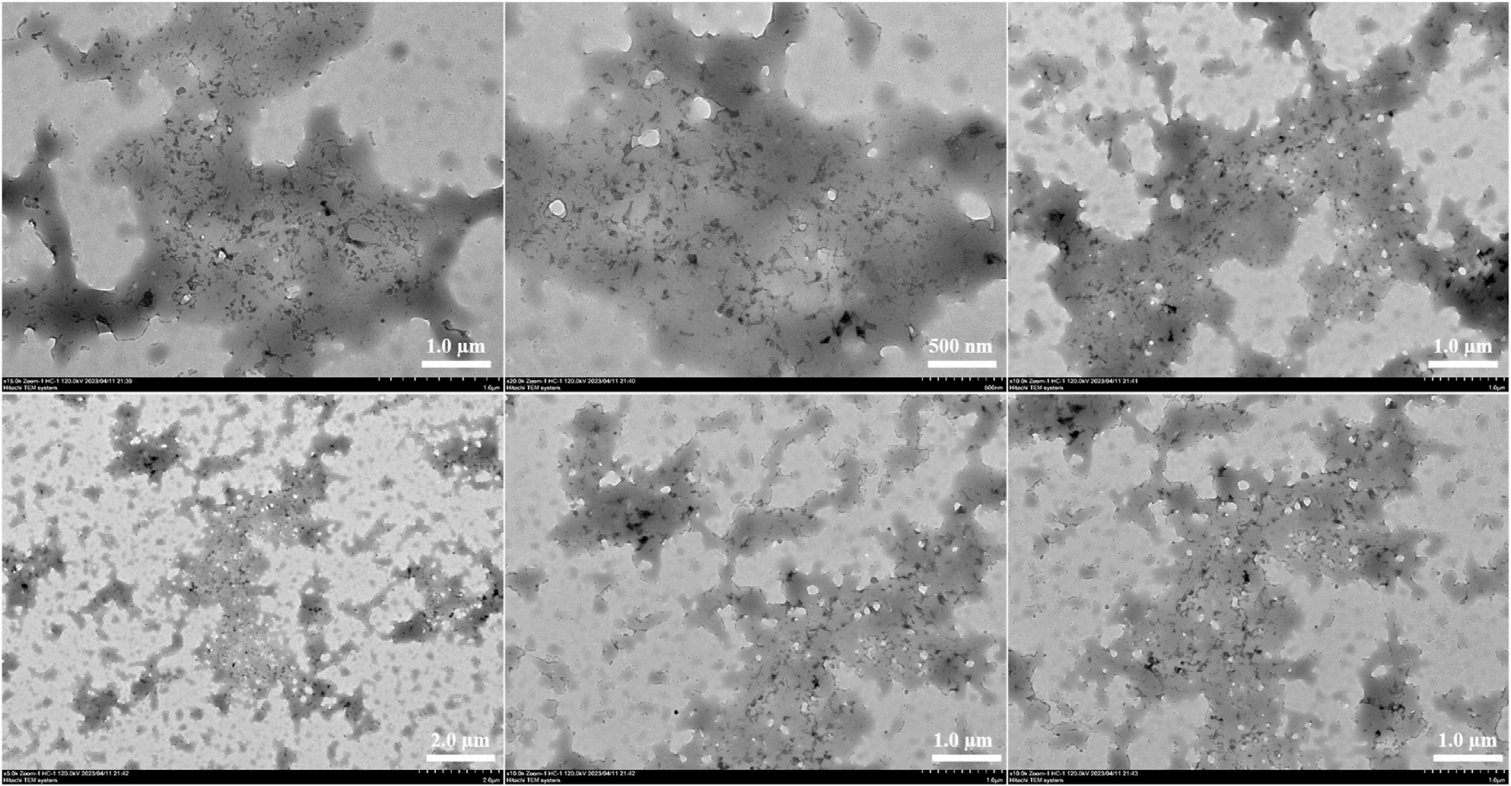
Transmission electron microscopy diagram of fluorescent complex FC-1.

Transmission electron microscopy (TEM) was used to investigate the internal morphology of FC-1 fluorescent complex. The FC-1 fluorescent complex was evenly distributed in the acrylic resin solution as an amorphous flocculent form, as seen in the figure. Additionally, the majority of fluorescent complexes are closely packed and entirely covered with acrylic resin. And extends out to enlarge its surface area like a porous model (Figure 4).

### Cell proliferation toxicity test (CCK8)

#### Cell imaging

**FIGURE 5:**
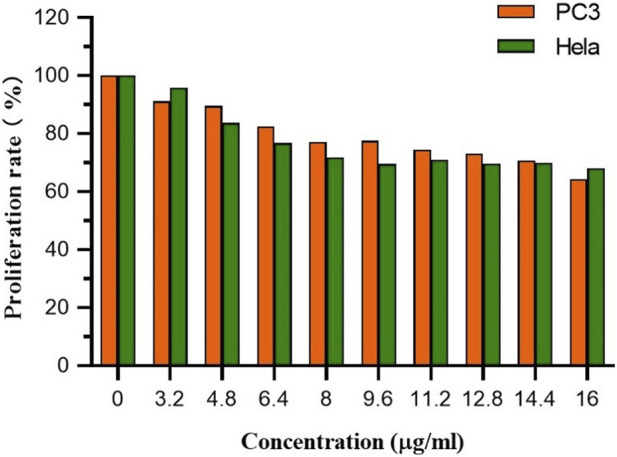
The proliferation rate of PC3 and Hela cells treated by different concentrations of FC-1.

CCK-8 was used to assess FC-1’s effects on PC3 and HeLa cell growth. In general, PC3 and HeLa cell growth is effectively inhibited by FC-1 chemicals. The proliferation rates of PC3 and HeLa treated with the substance were 64.30% and 68.06%, respectively, as shown in Figure 5 and Table 4, demonstrating good biological activity. Its inhibiting action also becomes quite apparent as concentration rises.

**TABLE 4:** The proliferation rate of PC3 and Hela cells treated by different concentrations of FC-1.

Concentration (μg/mL)	0	3.2 (%)	4.8 (%)	6.4 (%)	8 (%)	9.6 (%)	11.2 (%)	12.8 (%)	14.4 (%)	16 (%)
PC3	100.00	91.13	89.60	82.57	77.09	77.52	74.53	73.08	70.63	64.30
Hela	100.00	95.78	83.67	76.72	71.86	69.50	70.93	69.74	69.87	68.06

**FIGURE 6:**
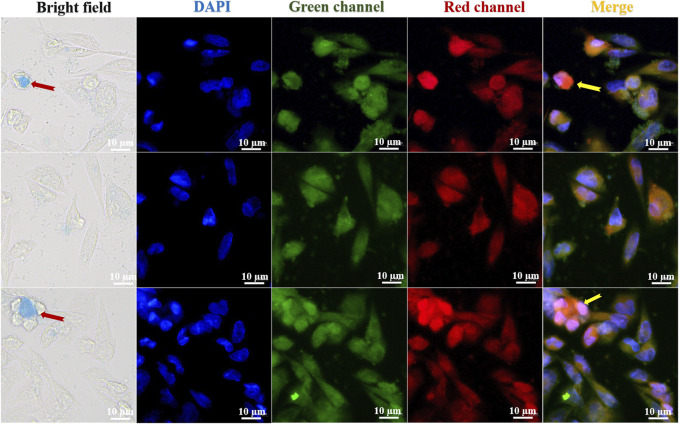
Fluorescence imaging of FC-1 fluorescent complexes in different channel HeLa cells.

Cell imaging was used to examine the stability and structure of the fluorescent compound in HeLa carcinoma cells to determine its selectivity and biocompatibility. With a fluorescent microscope, HeLa cell staining observed with a laser confocal microscope is plainly visible. The bright field, DAPI, green channel, red channel, and merge imaging are displayed in Figure 6. The FC-1 complex is biocompatible with HeLa cells and exhibits high selectivity. The blue visible specks in Figure 6 are FC-1 complexes, which are seen to enter and bind to tumor cells. In merge and overlay imaging, the staining impact of orange brilliant spots in tumor cells may be readily seen, while being less visible in DAPI, green channel, and red channel. This suggests that Coomassie Brilliant Blue G250 enhances and improves the selectivity and biocompatibility effects of isorhamnetin small molecule medicines by driving them into specific cells in the form of zwitterion.

## Conclusion

In this study, water-soluble Coomassie Brilliant Blue G250 and fat-soluble isorhamnetin were combined in a zwitterionic form by a one-pot method. Various types of acrylic resins were used as coating carriers to create the water-in-oil form, which produced the best results and increased the drug’s selectivity and biocompatibility to tumor cells. The application of many drugs may be possible with this approach. By combining drug excipients with zwitterionic technology from the inside out, a novel fluorescent composite drug was created. This drug not only resolved the issue of isorhamnetin’s insolubility and visual targeting, but also allowed the fluorescence complex to enter tumor cells in the form of nano-dispersion, greatly increasing the possibility of evaluating and using the drug’s activity both inside and outside the body. The CCK8 experiment’s findings demonstrated that FC-1 complex exhibited anti-tumor effect on PC-3 cells and Hela cells with increasing concentrations, and that tumor cell inhibition was 40%. This is because Coomassie Brilliant Blue G250 significantly reduces isorhamnetin’s outstanding inhibitory action. This is a challenging issue that will require further research to resolve. The aforementioned information can serve as a theoretical foundation for future studies and the creation of water-soluble isorhamnetin fluorescent complexes.

## Data Availability

The original contributions presented in the study are included in the article/Supplementary Material, further inquiries can be directed to the corresponding authors.
