# Supplementary material for: A porous form Coomassie brilliant blue G250-isorhamnetin fluorescent composite coated with acrylic resin for tumor cell imaging
Source: Front Chem. 2023 Sep 18;11:1260533. doi: 10.3389/fchem.2023.1260533 (PMC10544906; doi:10.3389/fchem.2023.1260533)
Supplement: Supplementary file 1 [file DataSheet1.docx]

**Supporting information**

**A porous form coomassie brilliant blue G250-isorhamnetin fluorescent composite coated with acrylic resin for tumor cell imaging**

Jiangpeng Hu^1*#^, Bo Teng^1#^, Zhipeng Xu^1^, Yuanye Wan^1^, and Guofan Jin^2*^

^1^Jiangsu University, Affiliated Peoples Hospital, Zhenjiang 212001, Jiangsu, P.R. China.

^2^School of Pharmacy, Jiangsu University, Zhenjiang 212013, P.R. China.

**
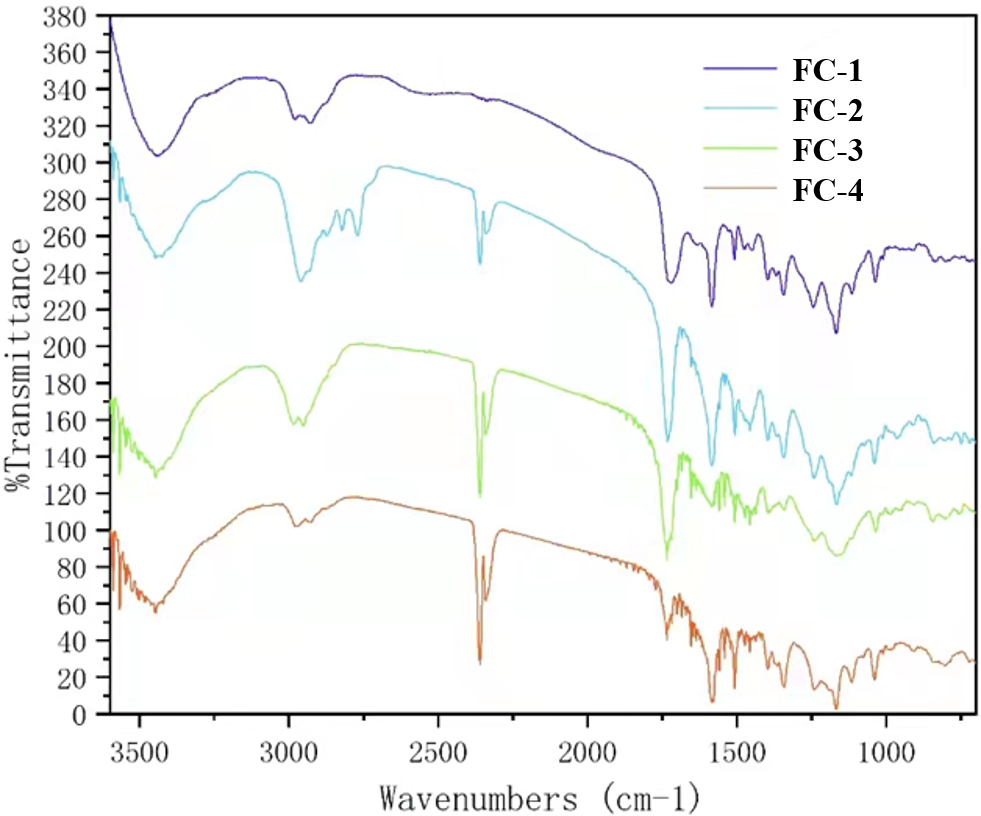
**

**Figure-s-1.** Infrared spectra of four kinds of fluorescent complexes.

**
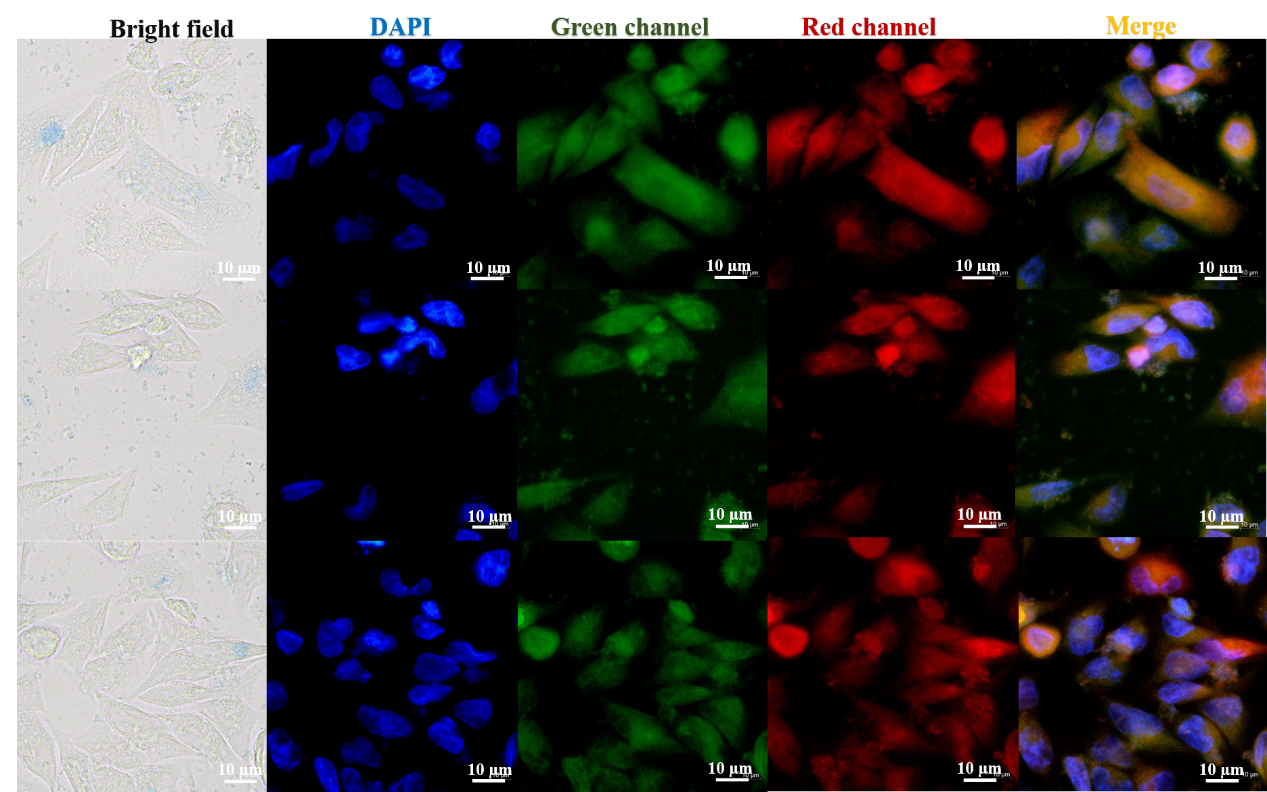
**

**Figure-s-2.** Fluorescence imaging of FC-1 fluorescent complexes in different channel HeLa cells.
